# Ultra-low profile solar-cell-integrated antenna with a high form factor

**DOI:** 10.1038/s41598-021-00461-w

**Published:** 2021-10-22

**Authors:** Ahmed Ali, Heesu Wang, Jaejin Lee, Yeong Hwan Ahn, Ikmo Park

**Affiliations:** 1 Department of Electrical and Computer Engineering, Ajou University, Suwon, 16499 Republic of Korea; 2 Department of Physics and Department of Energy Systems Research, Ajou University, Suwon, 16499 Republic of Korea

**Keywords:** Energy harvesting, Electrical and electronic engineering

## Abstract

This paper presents an ultra-low-profile copper indium gallium selenide (CIGS) based solar cell integrated antenna with a high form factor. A tiny slot was etched from the solar cell to develop an ultra-low-profile solar-cell-integrated antenna. This tiny slot increases the form factor due to the small clearance area from the solar cell. A ground-radiation antenna design method was applied in which lumped elements were employed inside the tiny slot for antenna operation. Another substrate was used under the solar cell for designing the feeding structure with lumped elements connected to the tiny slot using via holes. A prototype was fabricated and measured to verify the operation of a built-in solar-cell antenna and validate the simulated results. The measured results demonstrate that the solar-cell-integrated antenna covers the entire frequency range of the Industrial Scientific Medical band, from 2.4 to 2.5 GHz, with a maximum gain of 2.79 dBi and radiation efficiency higher than 80% within the impedance bandwidth range. Moreover, the proposed design has an ultra-low-profile structure of only 0.0046 λ_o_, where λ_o_ represents the free space wavelength at 2.45 GHz, and a high form factor of 99.1% with no optical blockage. The antenna and solar cell were designed to avoid affecting the performance of each other using the radio-frequency decoupler.

## Introduction

The ambition to build smart cities and societies relies highly on the large-scale deployment of the Internet of Things (IoT) and wireless sensor networks (WSNs)^1^. The employment of the IoT and WSNs on a large scale creates challenges, such as a sustainable power supply for sensors and end-node devices^2^. It is a cumbersome and challenging task to change the batteries of many IoT and WSN devices. Sometimes, changing batteries is impossible because these devices are deployed in inaccessible places. Moreover, batteries have problems, such as maintenance costs, hazardous waste, overheating, and charging losses. However, using renewable resources and green energy is encouraging, and the use of these resources has increased to counter environmental challenges^3^. One of the promising candidates for green energy is the solar cell^4^.

Recently, the use of solar cells has been increasing for wireless communication systems, especially for IoT and WSN applications where the power grid is not continually available^5^. Moreover, the overall efficiency and life span of IoT and WSN devices can be increased with solar cells. However, the device size is critical for some applications, and many different components compete for space to implement solar cells. For example, a solar cell was used with radio-frequency identification tags to increase the read range^6^, but a T-matched antenna was designed separately with a solar cell that takes extra space. One way to solve this problem is to integrate the antenna with a solar cell that results in a compact and low-weight dual-function device. Moreover, in recent years, we have seen extensive progress in the field of low-power electronics. This is in response to the high demand for low-power electronics in IoT and WSN applications, such as self-driving cars, smart homes, smart industry, smart grid, smart agriculture, security, and environmental monitoring. Also, various countries have started implementing the concept of IoT in flood and fire warning systems, intelligent transport systems (ITSs), and personal communication^7^. The advancement and progress of IoT in health care systems is developing a whole new concept of Healthcare IoT (H-IoT)^8,9^. The photovoltaic cell-integrated antenna can be employed as an on-board power supply for the above-mentioned low-power IoT applications. It can also be used with low-power wireless applications and sensor technologies. The idea of integrating solar cells with antennas dates to the 1990s for space applications^10^. Afterward, different methods and techniques have been presented to integrate solar cells with an antenna^11–18^.

A solar cell has been integrated with a dipole antenna for energy harvesting and wireless communications^19^, whereas a solar-cell-integrated antenna has been proposed for 2.4 GHz applications with a low profile structure^20^. Different antenna arrays have been integrated with a solar cell^21–25^. Amorphous silicon solar cells and dye-sensitized solar cells have been integrated with a microstrip slot antenna array^21,22^, whereas an antenna array has been integrated with multi-crystalline solar cells for low-power sensor applications^23^. The authors used an inverted-F antenna in an array, integrated vertically at the top of the solar cell^24^. A solar-cell-integrated optically transparent antenna array was presented where a meshed structure is used for designing antenna arrays^25^. Different solar-cell-integrated antennas have been proposed for CubeSats and satellite applications^26–28^. A circularly polarized (CP) meshed patch antenna was integrated with a solar cell for CubeSats and satellite applications^26^. An aperture coupled patch antenna^27^ and a CP transparent subarray antenna^28^ were integrated with the solar cell for CubeSats applications. However, the aforementioned solar-cell-integrated antenna design is large and has a complex design. Moreover, the solar cell and antenna work independently as two separate devices. A solar cell with a built-in antenna with a resonant slot structure has been proposed to solve this problem^29^. However, the antenna has a reduced form factor due to the resonant slot in the solar cell.

In this work, a solar cell with a built-in antenna with a high form factor is proposed for IoT and WSN applications. A commercially available copper indium gallium selenide (CIGS) solar cell is used, and a tiny slot is cut in the solar cell for resonance. The purpose of using a tiny slot structure is to increase the form factor. A ground-radiation antenna technique is used in which lumped elements are used in the ground plane for resonance^30–32^ to make the structure compact and low profile. The antenna is designed for 2.45 GHz Industrial Scientific Medical (ISM) band applications. The results show that the antenna has a form factor of 99% with excellent performance in terms of antenna characteristics.

## Solar cell with built-in antenna geometry

**Figure 1:**
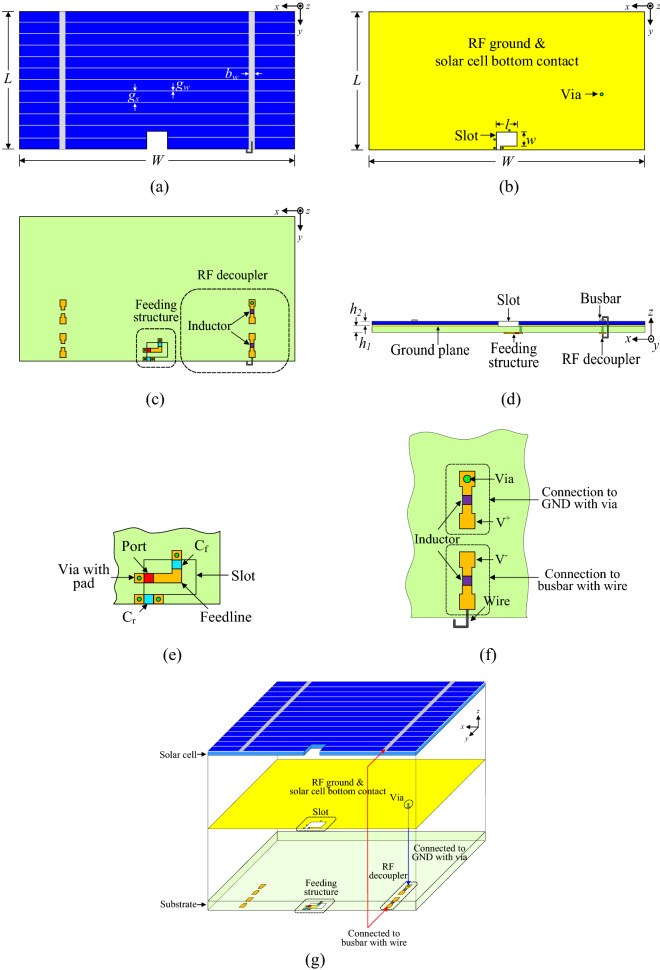
Geometry of the solar-cell-integrated antenna: (**a**) top view, (**b**) top view of bottom contact of the solar cell, (**c**) top view of the bottom side, (**d**) side view, (**e**) extended view of feeding structure with the slot, (**f**) extended view of RF decoupler circuit, and (**g**) 3D view.

A commercially available CIGS-based solar cell with the dimensions of *W* × *L* × *h*_1_ was used for the design. The solar cell consists of three layers. The top layer is a metallic trace of bus bar and grid with thickness of 0.001 mm. Each grid has a width of 0.1 mm, whereas the spacing between each grid is 1.5 mm. For the power collection, two bus bars were designed inside the grids. The width of each busbar is 1.5 mm, and the busbars were spaced apart at a distance of 20 mm. The second layer is thin layers of copper, indium, gallium, and selenide. The overall thickness of the CIGS layer is 0.004 mm. The bottom metallic contact of the CIGS solar cell comprises stainless steel with a thickness of 0.059 mm. The following design parameters were selected to model the CIGS solar cell in the EM simulator: the top and bottom metallic layers are modeled as a conductor with a conductivity of 5.8 × 10^7^ S/m, and the CIGS layer is modeled as a single dielectric layer with a dielectric constant and loss tangent of *ε*_*r*_ = 12.9 and *tanδ* = 0.0004, respectively. Figure 1a–g depict the top view of the solar cell, bottom view of the solar cell, top view of the bottom side of the second substrate, side view of the solar-cell-integrated antenna, extended view of feeding structure, extended view of the RF decoupler circuit, and 3D view of the solar-cell-integrated antenna, respectively.

For the antenna operation, a small slot was cut into the solar cell. The size of the slot is *l* × *w*, and it is located on the lower side of the solar cell. The purpose of using a small slot is to improve the form factor. The form factor is defined as the ratio of the area of the solar cell to the total area of the solar-cell-integrated antenna. It is well-known that the solar cell converts light energy to electrical energy through the photovoltaic effect. However, the collection of the light-generated carriers does not contribute to power generation without internal force; thus, the internal force induced by the doped layers is also required to operate the solar cell. Therefore, the p–n junction formed by doping is essential for power generation in a solar cell^33,34^. However, the doping layers have adverse effects on the antenna performance due to the conductivity in the solar cell. Therefore, a slot is also etched in the solar cell, reducing the effective area of the solar cell. A tiny slot is used to counter this problem so that the maximum solar cell area can be employed for power generation.

The design guidance and methodology of the proposed antenna were:

1. The antenna was designed by using the ground-radiation antenna technique. Compared to conventional antennas, ground-radiation antennas need a very small ground area clearance, and lumped elements are used inside the slot to create loop-type ground radiation.
2. Following the ground radiation antenna method, a small slot having an area of *l* × *w* was cut from the solar cell.
3. After that, two capacitors were added to create a resonance and feeding loop inside the slot. *C*_*r*_ was used to generate the resonance loop, while *C*_*f*_ was used to create the feeding loop. The capacitance values of both capacitors, *C*_*r*_ and *C*_*f,*_ were the same at 0.6 pF.
4. To connect the lumped elements and port with the slot, a second substrate with a thickness of 0.508 mm was used under the solar cell. The permittivity and loss tangent values of the second substrate are *ε*_*r*_ = 3.38 and *tanδ* = 0.0027, respectively. The feeding structure and capacitors were designed under the second substrate, and they are connected to the slot using via holes. Moreover, a radio-frequency (RF) decoupler circuit was also designed under the second substrate.

An RF decoupler circuit was designed to separate the functioning of the solar cell from the antenna. Two metallic traces were designed on the bottom side of the second substrate. Each metallic trace has an inductor of 56 nH that works as an RF choke. This RF choke suppresses the flow of alternating current (AC) toward the solar cell. In EM simulations, the size of each inductor was taken as 1 mm × 0.5 mm, and they were modeled as lumped elements. During simulations, it was found that varying the inductance value had only a minor effect on the antenna performance. The two metallic traces were designed to be near the bus bar for easy connections. A connecting wire was used to connect the busbar with the metallic trace, whereas the other metallic trace was connected to the bottom contact of the solar cell using via hole. The parameters for the final design were *h*_*1*_ = 0.063 mm, *h*_*2*_ = 0.508 mm, *W* = 50 mm, *L* = 25 mm, *l* = 3.7 mm, *w* = 3 mm, *C*_*f*_ = 0.6 pF, *C*_*r*_ = 0.6 pF, *g*_*s*_ = 1.5 mm, *g*_*w*_ = 0.1 mm, and *b*_*w*_ = 1.5 mm.

## Results and discussion

In ground radiation antennas, loop-type current modes are excited in the ground plane so that the ground plane can be used as a radiator. In the proposed design, a slot was etched, and two loops were created using lumped elements. The outer loop is the resonance loop and uses capacitor *C*_*r*_, while the inner loop is the feeding loop, and capacitor *C*_*f*_ is inserted in it. The resonance loop has a loop-type current that is excited by the feeding loop. The loop-type current in the resonance loop thus excites current modes in the ground plane that result in radiation. The proposed solar-cell-integrated antenna design has a − 10 dB impedance bandwidth from 2.38 GHz to 2.51 GHz covering the entire ISM 2.4 GHz band. The maximum gain within the impedance bandwidth was 2.91 dBi.

**Figure 2:**
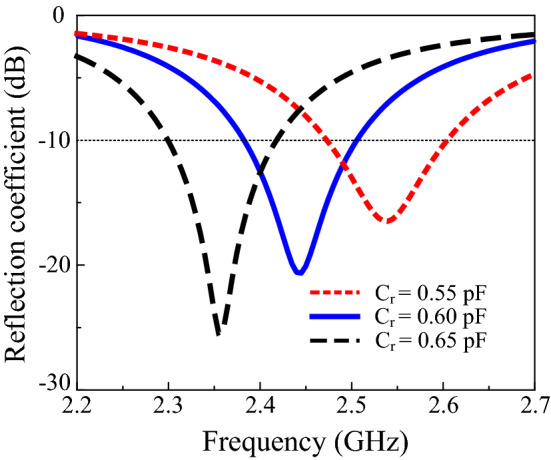
Reflection coefficients with varying *C*_*r*_.

**Figure 3:**
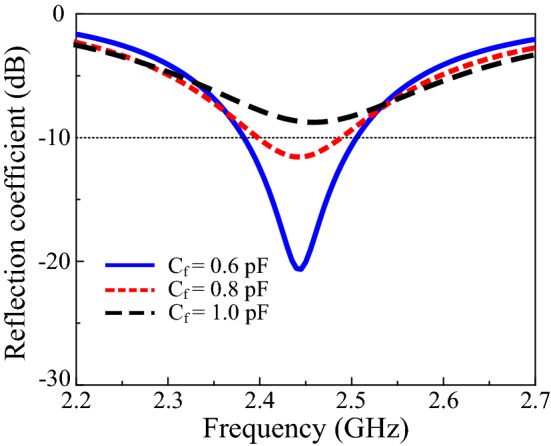
Reflection coefficients with varying *C*_*f*_.

The effects of the critical parameters of the proposed design were studied to understand the operation mechanism. Figure 2 illustrates the effects of various values of *C*_*r*_ on the reflection coefficient. The optimum value chosen for *C*_*r*_ was 0.6 pF. Changing the value of *C*_*r*_ from 0.55 pF to 0.65 pF shifts the resonance frequency from 2.44 GHz to 2.36 GHz, as illustrated in the figure. Increasing the *C*_*r*_ improves impedance matching, as it shifts to a lower frequency but does not affect the gain of the proposed design. The effects of *C*_*f*_ on the reflection coefficient are described in Fig. 3. The values for analysis of *C*_*f*_ were 0.6 pF, 0.8 pF, and 1 pF. Impedance matching improves with a decrease in *C*_*f*_; however, no change occurs in the gain and resonance frequency of the antenna. The optimum value selected for *C*_*f*_ was 0.6 pF.

**Figure 4:**
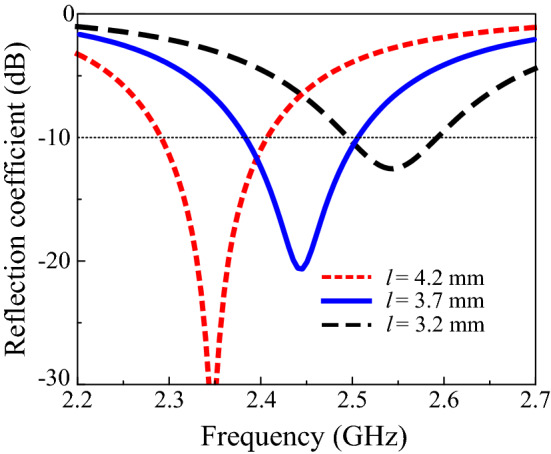
Reflection coefficients with varying slot lengths.

**Figure 5:**
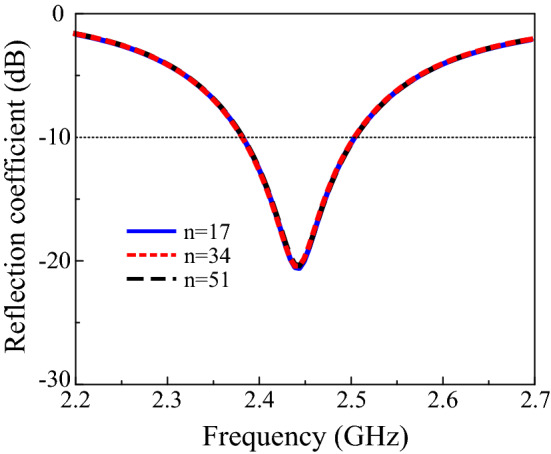
Reflection coefficients with different gridline numbers.

**Figure 6:**
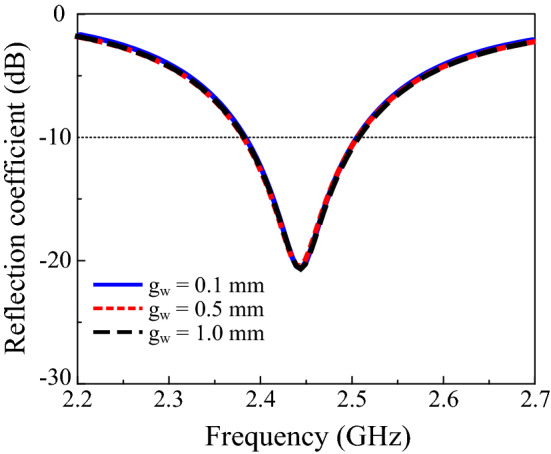
Reflection coefficients with different gridline widths.

Moreover, the effect of the slot length on the performance of the proposed design was studied. Figure 4 demonstrates that increasing the slot length from 3.7 mm to 4.2 mm shifts the resonance frequency from 2.44 GHz to 2.35 GHz. Furthermore, to investigate the effects of the solar cell on antenna operation, several grid lines and the width of the gridline were also studied. Increasing the number of gridlines and the width does not change the reflection coefficient of the proposed design. In addition, no significant change was observed in the gain of the proposed solar-cell-integrated antenna. Figures 5 and 6 depict the reflection coefficient of the proposed design with various gridline numbers and widths. An RF decoupler circuit was added between the solar cell and antenna to separate the functioning of the antenna from the solar cell. The simulation indicated that the addition of an RF decoupler circuit has a negligible effect on the performance of the solar-cell-integrated antenna. The performance of the proposed CIGS-based solar cell antenna design was compared with the state-of-the-art literature and is presented in Table 1. The comparison table reveals that the size of the solar-cell-integrated antenna reduces significantly while achieving form factor of 99.1%.

**Table 1:** Performance comparison of the proposed design with other solar cell antennas.

Refs	Size (λ_o_^3^)	IBW (%)	Gain (dBi)	Optical block (%)	Rad. type	Form factor (%)
^14^	1.10 × 1.10 × 0.076	4	4.1	0	Omni	25
^19^	1.10 × 1.32 × 0.22	52.1	9.87	0	Uni	12.5
^20^	1.6 × 1.2 × 0.024	15.5	8.55	0	Uni	51
^23^	1.27 × 1.27 × 0.056	3.35	3.5	5.3	Uni	97.3
^25^	4.6 × 4.6 × 0.06	6.8	20.14	12	Uni	100
^26^	0.62 × 1.03 × 0.06	4	3*	30	Uni	100
^29^	0.26 × 0.27 × 0.0054	31.49	2.8	0	Omni	90
Proposed	0.408 × 0.204 × 0.0046	5.71	2.79	0	Omni	99.1

*IBW* impedance bandwidth, *λ*_*o*_ free space wavelength at the center frequency of the IBW.

*Realized gain.

## Antenna measurement results

**Figure 7:**
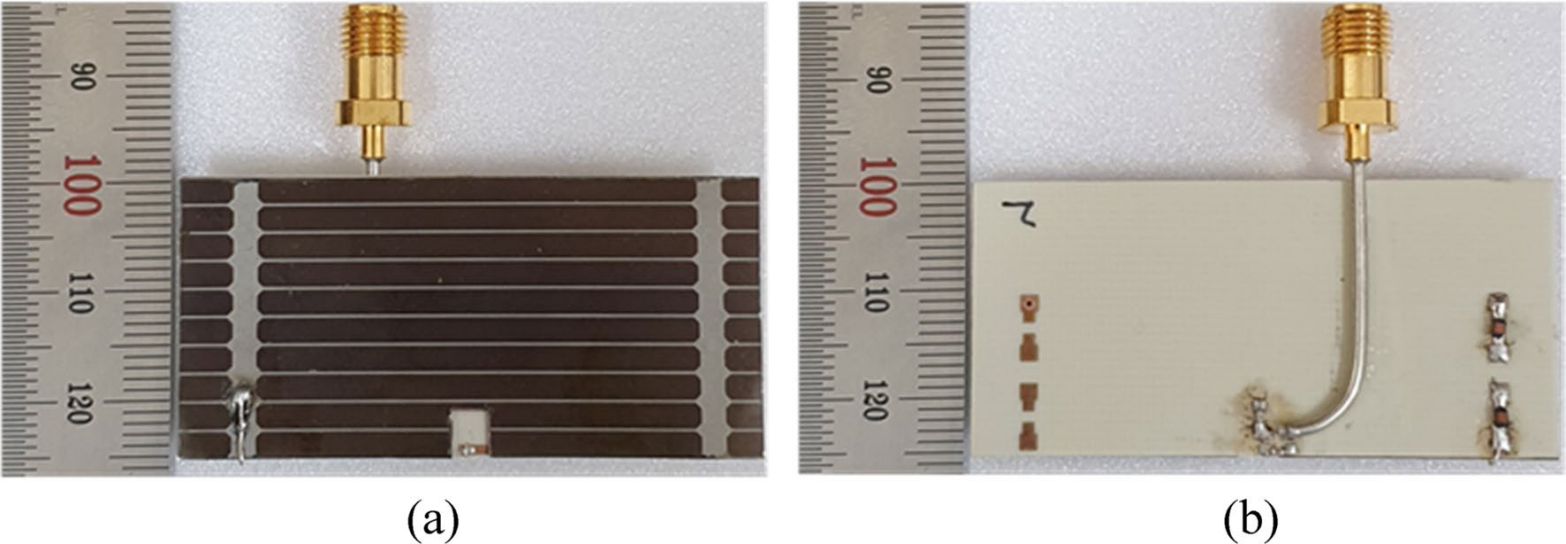
Photograph of the fabricated prototype: (**a**) top view and (**b**) bottom view.

**Figure 8:**
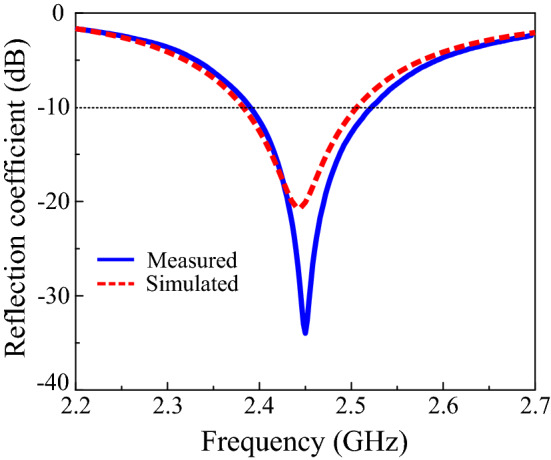
Reflection coefficient of the antenna.

The proposed solar-cell-integrated antenna was fabricated and measured to verify the simulation results. The photograph of the fabricated prototype is presented in Fig. 7. It can be seen in the figure that the fabricated prototype only has inductors on one side of the structure. This is because the solar cell worked well with only one inductor when coupled with the antenna. During simulations, it was also verified that using one vs. two chokes does not affect the antenna performance. A comparison of the simulated and measured results is displayed in Fig. 8. The measured − 10 dB impedance bandwidth was from 2.38 GHz to 2.52 GHz and is slightly wider than the simulated results, which were from 2.38 GHz to 2.51 GHz. Hence, the measured reflection coefficient also covers the entire 2.4 GHz ISM band.

**Figure 9:**
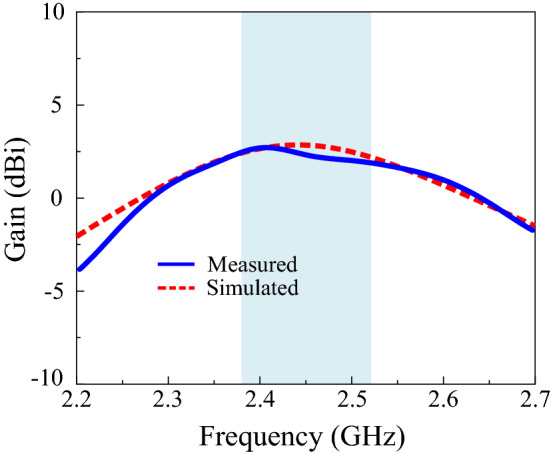
Gain of the antenna.

**Figure 10:**
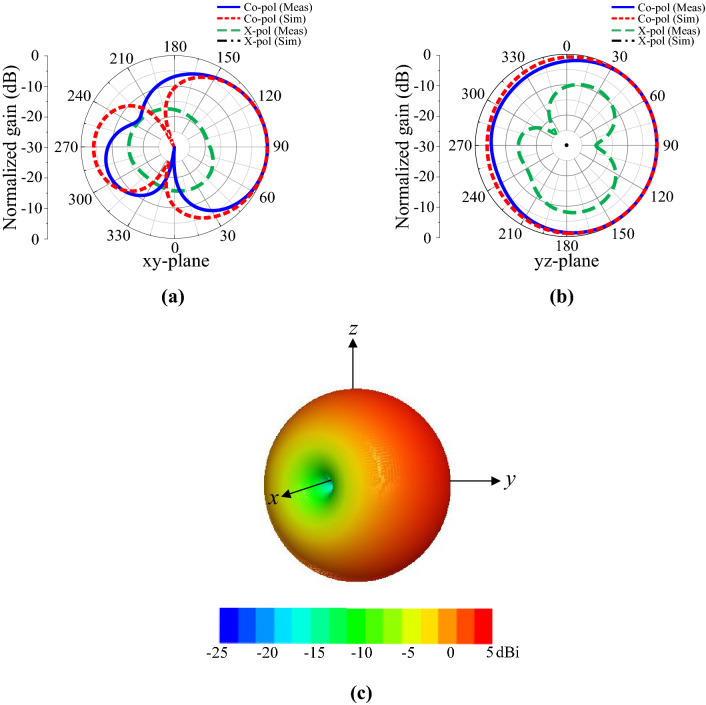
Normalized radiation patterns for the antenna at 2.45 GHz: (**a**) *xy*-plane (**b**) *yz*-plane, and (**c**) 3D radiation pattern.

The measured and simulated gain curves are illustrated in Fig. 9, where the measured gain is slightly lower than that in the simulated results. The maximum measured gain within the impedance bandwidth was 2.79 dBi. The difference between the measured and simulated gain curves can be attributed to fabrication and measurement inaccuracies. The 2D radiation patterns of the proposed design at 2.45 GHz are demonstrated in Fig. 10a,b. Normalized radiation patterns in the *xy*- and *yz*-planes are presented, as depicted in the figure. Overall, the radiation pattern of the antenna exhibits slight directional characteristics on the slot side. The surface current distribution was studied to analyze the radiation characteristics of the proposed antenna. The maximum current flows around the slot, which lies along the *y*-axis at the edge of the ground plane, resulting in a slight directional radiation pattern, as illustrated in Fig. 10c. Moreover, the simulated radiation patterns in the *xy-* and *yz*-planes are almost identical to the measured results, as presented in Fig. 10.

## Methods

The proposed antenna structure was analyzed and optimized using a full-wave electromagnetic wave high-frequency structure simulator. For the fabrication, a 0.508 mm thick Rogers sheet with a dielectric constant of 3.38 and a loss tangent of 0.0027 was used and standard printed circuit board technology was employed to design the feeding structure on the bottom side. After fabrication, the capacitors and inductors were connected using soldering. Finally, a slot was cut from the CIGS solar cell and was attached to the Rogers substrate.

A Rohde and Schwarz ZVA67 network analyzer was used to measure the reflection coefficient. The network analyzer was calibrated before measuring the reflection coefficient of the fabricated prototype. The radiation characteristics of the proposed antenna were measured in a full anechoic chamber. In the anechoic chamber, a fixed-horn antenna was used as a transmitter, whereas a fabricated antenna was employed as a receiver and rotated from − 180° to 180°. The distance between the transmitter and receiver was 10 m.

## Conclusion

This work used a CIGS-based solar cell as an antenna, making a single dual-functional device. A small slot was cut in the solar cell, and lumped elements were used with the slot for resonance to obtain the antenna functionality from a solar cell. The feeding structure and lumped elements were designed under the second substrate, which was also used to support the solar cell. An RF decoupler circuit was used to separate the operation of the antenna from the solar cell. The built-in solar-cell antenna has a very compact size of 50 mm × 25 mm × 0.571 mm (0.408 λ_o_ × 0.204 λ_o_ × 0.0046 λ_o_ at 2.45 GHz). The proposed design covers the ISM 2.45 GHz band with a maximum gain of 2.79 dBi and radiation efficiency higher than 80% within the impedance bandwidth. Furthermore, the solar cell had a form factor of 99%, achieved with the tiny slot area of only 3 mm × 3.7 mm in the solar cell. Finally, the measured results agree well with simulated results; thus, the proposed built-in solar-cell antenna is a promising candidate for clean energy and green communication.
